# Radiologically determined orthodontically induced external apical root resorption in incisors after non-surgical orthodontic treatment of class II division 1 malocclusion: a systematic review

**DOI:** 10.1186/s40510-014-0048-7

**Published:** 2014-07-23

**Authors:** Long D Tieu, Humam Saltaji, David Normando, Carlos Flores-Mir

**Affiliations:** School of Dentistry, University of Alberta, Edmonton, Alberta T6G 1C9 Canada; Department of Orthodontics, Faculty of Dentistry, Federal University of Para, Belém, 66055-090 Brazil; Edmonton Clinic Health Academy (ECHA), University of Alberta, Room 5-528, 11405-87 Ave, Edmonton, T6G 1C9 Canada

**Keywords:** Root resorption, Systematic review, Class II malocclusion

## Abstract

This study aims to critically evaluate orthodontically induced external apical root resorption (OIEARR) in incisors of patients undergoing non-surgical orthodontic treatment of class II division 1 malocclusion by a systematic review of the published data. An electronic search of two databases was performed; the bibliographies of relevant articles were also reviewed. Studies were included if they examined the amount of OIEARR in incisors produced during non-surgical orthodontic treatment of individuals with class II division I malocclusion in the permanent dentition. Individuals had no previous history of OIEARR, syndromes, pathologies, or general diseases. Study selections, risk of bias assessment, and data extraction were performed in duplicate. Eight studies of moderate methodological quality were finally included. An increased prevalence (65.6% to 98.1%) and mild to moderate severity of OIEARR (<4 mm and <1/3 original root) were reported. No sex difference in root resorption was found. For the maxillary incisors, there was no evidence that either the central or lateral incisor was more susceptible to OIEARR. A weak to moderate positive correlation between treatment duration and root resorption, and anteroposterior apical displacement and root resorption was found. Current limited evidence suggests that non-surgical comprehensive orthodontic treatment to correct class II division 1 malocclusions causes increased prevalence and severity of OIEARR the more the incisor roots are displaced and the longer this movement takes.

## Review

### Introduction

Orthodontically induced external apical root resorption (OIEARR) is a relatively common iatrogenic problem that has challenged orthodontists for many years. Histologic studies have reported greater than 90% occurrence of OIEARR [1–4], whereas radiographic evaluation studies reported between 48% and 66% occurrence [5–8]. OIEARR is usually less than 2.5 mm [9–12] when assessed by panoramic or periapical radiographs and is typically classified as mild to moderate with minor clinical significance. On rare occasions, severe resorption exceeding 4 mm, often classified as loss of more than a third of the original root length, has been reported in 1% to 10% of treated teeth [5,7,13–16].

The etiology of OIEARR is unclear with various studies reporting 7% to 15% of untreated patients present with non-orthodontically induced external apical root resorption prior to orthodontic treatment [5,17]. Individuals vary in their susceptibility to OIEARR with various factors such as tooth root morphology [10], length [10], genetics [18], and chronological age [19]. There are also a number of reported orthodontic treatment-related risk factors suggested in the literature such as treatment duration [13,20], magnitude of applied force [4], and amount of apical movement [20].

There have been a number of previous literature reviews [21–24], a systematic review [25], and a meta-analysis [26] reporting root resorption and OIEARR. While these reports looked at OIEARR, they did not specifically address root resorption of maxillary and mandibular incisors in non-extraction or extraction treatment of class II malocclusions. Class II division 1 malocclusions affect approximately 1/3 of the North American population [27]. There are reports of a 20-fold increase in the risk of severe root resorption of maxillary incisors if their roots were forced against the cortical plate during treatment [28]. This is likely to occur when attempting to camouflage a skeletal problem, as seen in class II correction that has not been surgically treated. Since root resorption risk varies from individual to individual, it is important to critically assess the different treatment techniques in order identify the presence of specific factors that may be identified to help reduce the incidence of this problem.

As such, the purpose of this systematic review is to critically analyze the available scientific literature regarding OIEARR to maxillary and mandibular incisors during non-surgical orthodontic treatment (extraction and non-extraction) in class II division I malocclusions.

### Materials and methods

Reporting of this systematic review was performed in accordance with the PRISMA statement for reporting systematic reviews of health sciences interventions [29].

#### PICO

Among class II division 1 malocclusion individuals, does the current non-surgical treatment mechanics produce radiographically determined OIEARR in incisors?

#### Data sources and searches

Comprehensive searches up to 20 July 2013 were conducted using the following electronic bibliographic databases: PubMed (1966 to July 2013, week 3) and MEDLINE (OvidSP) (1980 to 2013, week 28). The terms used for this literature search were ‘root resorption’ , ‘root shortening’ , ‘malocclusion’ , ‘class II’ , and ‘orthodontics’. The initial search strategy was designed for PubMed (Table 1) and later adapted to Medline. From the selected articles, hand searches were subsequently performed on the reference lists. No restrictions were applied regarding publication year or language. When additional information was needed, efforts were made to contact the authors.

**Table 1:** **Search strategies and results from different electronic databases**

**Database**	**Keywords**	**Results**
PubMed 1966 to July 2013, week 2	(Root* Resorption OR Root* Shortening) AND (Orthodontic* OR Class II OR Malocclusion*)	302
Ovid MEDLINE(R) 1946 to July Week 2 2013	((Resorption.ab or resorption.in or resorption.kf or resorption.kw or resorption.nm or resorption.ot or resorption.ti) OR (Shortening.ab or shortening.in or shortening.kf or shortening.kw or shortening.ot or shortening.ti)) AND ((Exp Orthodontic brackets or orthodontic*.mp or exp Orthodontic appliances or exp orthodontic extrusion or exp orthodontics space closure or exp orthodontic appliance design or exp Orthodontic retainers or exp Orthodontic Appliance, functional or exp orthodontic anchorage procedures or exp ‘index of orthodontic treatment need’ or exp orthodontic wire or exp orthodontics appliances, removable) OR (Malocclusion*.mp or exp Malocclusion or exp Malocclusion Angle Class II))	1,529
Total electronic databases searches		1,831
Duplicates		109
Final		1,722

*Truncation of words.

#### Study selection

Appropriate studies to be selected met the following pre-defined inclusion-exclusion criteria:

- *Population:* individuals with class II division 1 malocclusion. Individuals should have no history of root resorption, syndromes, pathologies, or general diseases. Only human studies were eligible with no restrictions applied regarding sex
- *Intervention:* a non-surgical orthodontic treatment of Class II division 1 malocclusion: either extraction treatment (bicuspids extraction on the upper and/or lower arch) or non-extraction treatment (e.g., functional therapy by removable or fixed appliances with class II elastics)
- *Comparison:* before and after treatment or extraction versus non-extraction treatment or another equivalent intervention (non-treated control)
- *Outcome:* root resorption evaluated by the root lengths of maxillary and mandibular teeth assessed using radiographic imaging (e.g., periapical, cone-beam computer tomography images)
- *Study design:* randomized, non-randomized controlled trials, clinical trial, prospective, and retrospective reports were included. Case reports or case series studies were not included

In the first step of the review process, two reviewers (LT and HS) independently reviewed the list of titles and abstracts for inclusion. Once potentially adequate abstracts were selected, full articles were retrieved in a second final selection process. If the abstract was judged to contain insufficient information for a decision of inclusion or exclusion, the full article was obtained and reviewed before a final decision was made. In the second phase of selection, eligibility criteria were applied to the full articles. Any discrepancies in the inclusion of articles between reviewers were addressed through discussion until consensus was reached.

#### Risk of bias

Two reviewers evaluated the methodological and reporting quality of the finally selected reports; discrepancies were resolved by discussion until consensus was reached. The following quality items were used to assess the methodological quality and risk of bias in the studies [30,31]: eligibility criteria, adequacy of sample size, reporting of randomization, reporting of blinding, avoiding selective reporting, description of intervention details, description of outcome measures, description of adverse effects, and adequacy of data analysis.

#### Data extraction

Data was extracted for each of the selected studies based on the following outcomes: study design, sample size, and age at start of treatment, whether there were extractions, and method of class II division 1 treatment. Study demographics including publication year and country where study was conducted were also collected. Data extraction was done by two investigators (LT and HS). Any discrepancies were resolved by discussion until an agreement was reached.

#### Data synthesis

Data was planned to be pooled in order to provide an estimate of the effectiveness of the interventions planned for the studies reporting the same outcome measures. Evaluation of clinical heterogeneity was planned to be performed by examining various characteristics of the finally selected reports, such as the dissimilarity between the different types of interventions, outcomes, and patients. A qualitative analysis was planned for any intervention where there was an insufficient clinically homogeneous trial.

### Results

#### Study selection

**Figure 1:**
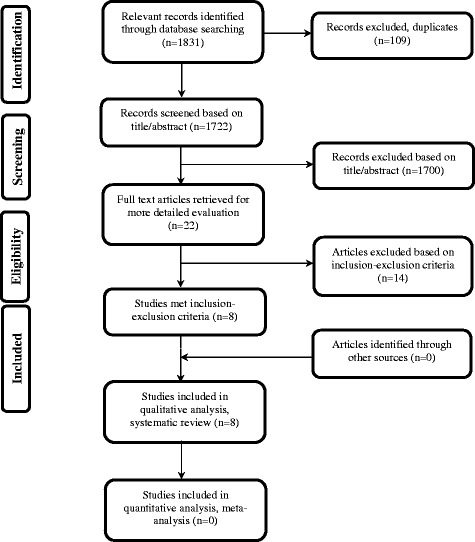
**Flow diagram of data search according to PRISMA [** 29 **].**

The search yielded 1,831 potential studies for inclusion from different electronic databases (Tables 1, 2, 3). Full texts of 22 journal articles were retrieved for further evaluation. Ultimately, nine papers fulfilled the inclusion-exclusion criteria, however eight [32–39] studies were included since one author had two [37,40] different publications using the same sample group. No additional studies were identified through the reference list search, and an update search revealed no additional studies. A flow diagram of the data search can be seen in Figure 1. The excluded studies and the reasons for their exclusion can be found in Table 4.

**Table 2:** **PubMed selection**

**Search number**	**Search**	**Items found**
1	Root* Resorption	4,502
2	Root * Shortening	453
3	Orthodontic*	49,955
4	Class II	79,607
5	Malocclusion*	27,701
6	No. 1 OR no. 2	4,920
7	No. 3 OR no. 4 OR no. 5	135,322
8	No. 6 AND no. 7	302

*Truncation of words.

**Table 3:** Number of hits from the PubMed search

**Search number**	**Search**	**Items found**
1	Resorption.ab or resorption.in or resorption.kf or resorption.kw or resorption.nm or resorption.ot or resorption.ti	37,091
2	Shortening.ab or shortening.in or shortening.kf or shortening.kw or shortening.ot or shortening.ti	41,579
3	Exp Orthodontic brackets or orthodontic*.mp or exp Orthodontic appliances or exp orthodontic extrusion or exp orthodontics space closure or exp orthodontic appliance design or exp Orthodontic retainers or exp Orthodontic Appliance, functional or exp orthodontic anchorage procedures or exp ‘index of orthodontic treatment need’ or exp orthodontic wire or exp orthodontics appliances, removable	44,406
4	Malocclusion*.mp or exp Malocclusion or exp Malocclusion Angle Class II	30,472
5	No. 1 OR no. 2	78,546
6	No. 3 OR no. 4	58,589
7	No. 5 AND no. 6	1,529

Duplicates - 109.

Search number overall − duplicates = 1,722.

**Table 4:** **Articles not selected from the initial abstract selection list and reasons for exclusion**

**Article**	**Reason for exclusion**
Goldson and Henrikson 1975 [41]	1
Lee et al. [42]	1
McNab et al. [43]	1
Alwali et al. [44]	4
Brin et al. [45]	3
Segal et al. [46]	2
Nasiopoulos et al. [47]	4
Janson et al. [48]	1
Huang et al. [49]	1
Weltman et al. [50]	2
Sunku et al. [51]	1
Kinzinger et al. [52]	4
Wahab et al. [53]	4

1, Mixed data (class II malocclusion data mixed with other malocclusion); 2, review (literature or systematic review); 3, mixed trauma data; 4, unrelated data.

**Table 5:** **Additional included study characteristics**

**Article**	**X-ray**	**Validity**	**Precession**	**Extraction**	**Class II treatment**
					**Mechanics and additional treatment information**
DeShields 1969 [32]	PA	✓	✓	No	All class II division 1 malocclusion
					Edgewise appliance
					Headgear (HP, M, L) or class II elastics
					RR assessed using PA
					AP and vertical root movement assessed using Ceph
					Compared to similar untreated pts
Hollender et al. [33]	PA	✓	✓	Yes	All class II division 1 malocclusion
				Upper 14/24	Edgewise appliance
					RR assessed using PA
Eisel et al. [34]	PA	✓	✓	Yes 64%	48% Edgewise
				No 36%	9% Only functional
					43% Combined
Reukers et al. [35]	PA	✓	✓	Yes and no	Class II malocclusion (divisions 1, 2, and subdivisions)
				If extractions were done could be 2, 3, or 4 premolars extracted	Edgewise vs. straight wire
					Edgewise - sliding
					Straight wire - loops
					Class II elastics
					0.022-in. Standard edgewise slot
					0.018-in. Straight wire edgewise slot
					Roth prescription
					Not clear how many cases were treated with extractions/non-extraction
					RR assessed using PA
Taner et al. [36]	Ceph	✘	✘	Yes	Class II division 1 malocclusion
				Four 1st premolar	0.018-in. Edgewise slot
					RR, AP, and vertical root movement assessed using Ceph
					OJ corrected with controlled tipping of upper incisors
Mavragani et al. [40]	PA	✓	✓	Yes	Class II division I malocclusion
Mavragani et al. [37]				At least 14/24 or upper bicuspid with lower 4s or upper bicuspid with lower 5s	0.018-in. Standard or straight wire edgewise slot
					Edgewise vs. straight wire
					RR assessed using PA
					Compared to similar untreated pts
Liou and Chang [38]	PA	✓	✓	Yes	Class II division I malocclusion
				Mx 14/24	Edgewise appliance
					En-masse Mx anterior retraction and FFA vs. FFA
					Anterior retraction using NiTi coils
					RR assessed using PA
Martins et al. [39]	PA	✓	✓	Yes	Class II malocclusion
				(2 Mx PM or 4 PM)	0.022-in. edgewise slot
					HG and class II elastics (if necessary)
					RR assessed using PA

PA, Postero-Anterior Cepahalometric X-ray; HP, High-pull; M, Medium -pull; L, low-pull; RR, Root resorption; AP, Antero-Posterior; Ceph, Lateral Cephalometric X-ray; OJ, Overjet; FFA, Full Fixed Appliances; NiTi, Nickel-Titanium; HG, Headgear.

#### Study characteristics

A summary of the methodological data and study results can be found in Tables 5, 6 and 7. Seven of the studies were retrospective studies and one was prospective. Seven studies were written in English and one was written in German.

**Table 6:** **Description of selected studies**

**Article**	**Study design**	**Country**	**Sample size**	**Age at** ***T***_**1**_ **(years)**	**Extraction**	**Class II treatment**
DeShields [32]	RS	USA	52 (24 M/28 F)	M = 12.6/F = 12.2	No	Headgear or class II elastics
				Group = 12.4 ± 0.9 Y		
Hollender et al. [33]	RS	Sweden	12 (3 M/9 F)	Group 1 = 3 Y 3 M	Yes	Edgewise
					Upper 14/24	
Eisel et al. [34]	RS	Germany	44	Group 1 = 4.7 (7.1)	Yes 64%	48% Edgewise
					No 36%	9% Only functional
						43% Combined
Reukers et al. [35]	PS	Holland	Started 149 (64 M/85 F)	Group 1 = 2 Y 4 M ± 1 Y 2 M	Yes and no	29 Edgewise
					If extractions were done could be 2, 3, or 4 premolars extracted	32 Straight wire Class II elastics
			Finished 61			
			Excluded 2 - moved 7 - early debond 79 - poor radiographs			
Taner et al. [36]	RS	Turkey	27	Group 1 = 3.6 ± 2.5 Y	Yes	Edgewise
					4 bicuspids	
Mavragani et al. [40] Mavragani et al. [37]	RS	Norway	80	Edgewise 13.8 ± 0.7 Y	Yes	40 Edgewise
			Edgewise (22 M/18 F)	Straight wire	At least 14/24 or upper bicuspid with lower 4s or upper bicuspid with lower 5s	40 Straight wires
				13.1 ± 0.7 Y		
			Straight wire (20 M/20 F)			
Liou and Chang [38]	RS	Taiwan	50	Group I = 26.5 ± 5.5 Y	Yes	I-30 Minscrew + FFA
			Group I (0 M/30 F)	Group II = 22.5 ± 1.6 Y	Mx 14/24	II-20 FFA
			Group II (4 M/16 F)			
Martins et al. [39]	RS	Brazil	28	I = 13.4 ± 2.4 Y	Yes	Edgewise
			I (16 M:12 F)		(2 Mx PM or 4 PM)	Headgear and class II elastics (if necessary)
			II N/A mixed data			

RS, retrospective study; PS, prospective study; RCT, randomized controlled trial; Y, years; M, males; F, females. M, Months; FFA, Full Fixed Appliances; N/A, Not Applicable; PM, premolar.

**Table 7:** **Descriptive statistics of selected studies**

**Article**	**Treatment duration (months)**	**X-ray**	**Root resorption (RR)**	**RR - specific tooth and severity**	**Additional information**
DeShields [32]	M = 20.5	PA	51/52 cases had resorption in at least 1 Mx incisor	Tooth 12^a^	Treatment time edgewise (months)
	F = 22.5			Grade 0 - 1/52	Male = 11.6
	G = 21.6 ± 5.2		Severity^a^	Grade 1 - 4/52	Female = 10.1
			Grade 0 - 12/208	Grade 2 - 23/52	Mean = 10.8 ± 5.7
			Grade 1 - 24/208	Grade 3 - 21/52	Headgear time (if used) (months)
			Grade 2 - 82/208	Grade 4 - 3/52	Male = 16.5
			Grade 3 - 79/208	Tooth 11^a^	Female = 17.0
			Grade 4 - 11/208	Grade 0 - 4/52	Mean = 16.8 ± 7.6
			Grade 5 - 0/208	Grade 1 - 7/52	Class II elastics (if used) (months)
			Gender-severity^a^	Grade 2 - 19/52	Male = 7.5
			4 M - grade 2	Grade 3 - 17/52	Female = 5.2
			17 M - grade 3	Grade 4 - 5/52	Mean = 6.3 ± 5.6
			3 M - grade 4	Tooth 21^a^	
			1 F - grade 1	Grade 0 - 3/52	
			7 M - grade 2	Grade 1 - 5/52	
			17 F - grade 3	Grade 2 - 26/52	
			3 F - grade 4	Grade 3 - 17/52	
				Grade 4 - 1/52	
				Tooth 22^a^	
				Grade 0 - 4/52	
				Grade 1 - 8/52	
				Grade 2 - 14/52	
				Grade 3 - 24/52	
				Grade 4 - 2/52	
Hollender et al. [33]	Mean = 18	PA	Grade I or II RR^b^	Tooth (grade 1 or 2 RR)^b^	Mx anterior teeth most affected
			60/120 Teeth	16 - 3/12	48/60
			Severity	15 - 3/12	Lateral incisor
			Grade 1 - 53/60	13 - 5/12	22/24
			Grade 2 - 7/60	12 - 11/12	No grade 3 resorption
				11 - 7/12	
				21 - 6/12	
				22 - 11/12	
				23 - 8/12	
				25 - 3/12	
				26 - 3/12	
Eisel et al. [34]	38 ± 20 (total sample)	PA	Mean four upper incisors:	Not described	Only 29 patients had periapicals to quantify RR. No explanation why only these ones
			Up to 1 mm, 21 individuals		
			Between 1 and 2 mm, 6 individuals		
			Between 2 and 3 mm, 1 individual		
			More than 3 mm, 1 individual		
			Maximum for either upper incisor		
			Up to 1 mm, 12 individuals		
			Between 1 and 2 mm 6 individuals		
			Between 2 and 3 mm 6 individuals		
			More than 4 mm 5 individuals		
					RR dx through Linge and Linge method [12]
Reukers et al. [35]	Overall = 20.4 ± 6.0	PA	Mean degree of resorption		Statistical test showed no difference in root resorption between straight wire and edgewise
	Straight wire = 21.6 ± 4.8				
			Overall – 7.8 ± 6.9%		
	Edgewise = 19.2 ± 6.0		Straight wire - 8.2 ± 6.4%		
			Edgewise - 7.5 ± 7.6%		Study only focused on root resorption of Mx central incisors
			Prevalence		
			Overall - (40/61) 65.6%		
			Straight wire - (24/32) 75%		
			Edgewise - (16/29) 55%		
Taner et al. [36]	28.1 ± 9.0	Ceph	Mean RR	N/A	N/A
			2.1 ± 1.6 mm		
Mavragani et al. [40]	N/A	PA	Same data as 2002	Same data as 2002	Same data as 2002
Mavragani et al. [37]	N/A	PA	Tooth/median	Mean RR	Root elongation was noted for 50/280 teeth
			Control	12 - 1.86 ± 0.26 mm	
			12 - 17.03 mm	11 - 1.82 ± 0.26 mm	Age at *T*_1_ was significantly higher among patients showing root shortening of lateral incisors than those showing root elongation (*p* < 0.05)
			11 - 16.79 mm	21 - 1.93 ± 0.25 mm	
			21 - 16.69 mm	22 - 1.78 ± 0.33 mm	
			22 - 17.48 mm	Shortened roots	Roots that were incompletely developed before treatment reached a significantly greater length than those that were fully developed at the *T*_1_
			Shortened	12 - 59/72 teeth	
			12 - 14.55 mm	11 - 60/72 teeth	
			11 - 15.32 mm	21 - 58/67 teeth	
			21 - 15.30 mm	22 - 53/69 teeth	
			22 - 13.77 mm		
			Elongated	Elongated roots	
			12 - 17.36 mm	12 - 13/72 teeth	
			11 - 17.56 mm	11 - 12/72 teeth	
			21 - 15.52 mm	21 - 9/67 teeth	
			22 - 16.85 mm	22 - 16/69 teeth	
Liou and Chang [38]	En-masse	PA	Group I	Group I	Group I (ANB 7.1° ± 1.9°)
	(Group I) 28.3 ± 7.3				
	FFA		16% to 20% (2.5 to 2.8 mm)	12 - 20.0 ± 7.3% (2.7 ± 1.0 mm)	Group II (ANB 3.2° ± 2.9°)
	(Group II) 22.7 ± 5.0		Group 2		Apical RR of Mx central incisor was significantly correlated to the duration of treatment (*p* = 0.026) but not to the amount of en-masse retraction, intrusion, or palatal tipping of Mx incisors
			13.4% to 14.4% (2.1 to 2.3 mm)	11 - 19.6 ± 6.6% (2.8 ± 1.0 mm)	
				21 - 16.8 ± 8.8% (2.5 ± 1.4 mm)	Mx lateral incisors was significantly greater in group I than in group II
				22 - 16.0 ± 9.2% (2.5 ± 1.5 mm)	
				Group II	
				12 - 14.4 ± 7.3% (2.1 ± 1.4 mm)	
				11 - 14.4 ± 8.5% (2.3 ± 1.7 mm)	
				21 - 13.6 ± 7.6% (2.1 ± 1.5 mm)	
				22 - 13.4 ± 7.3% (2.1 ± 1.3 mm)	
Martins et al. [39]	28.0 ± 9.4	PA	RR severity^c^		
			0 = 0/112 (0%)		
			1 = 19/112 (16.96%)		
			2 = 39/112 (34.83%)		
			3 = 47/112 (41.96%)		
			4 = 7/112 (6.25%)		

^a^Grade 0 - no resorption, grade 1 - possible resorption (some indistinctness to apical outline), grade 2 - definite resorption (apical outline was definitely irregular, but the root was not shortened or blunted), grade 3 - mild apical blunting (<3 mm), grade 4 - moderate apical blunting (>3 mm but <1/3 root length, grade 5 - severe blunting (>1/3 of the root length loss); ^b^0 - no visible resorption, 1 - apical resorption ≤2 mm, 2 - apical resorption >2 mm ≤1/3 of the root length, 3 - >1/3 of the root length; ^c^0 - no root resorption, 1 - mild resorption, normal length, and only displaying irregular contour, 2 - moderate resorption, small area of root loss with the apex exhibiting an almost straight contour, 3 - accentuated resorption, loss of almost 1/3 root length, 4 - extreme resorption, loss of more than 1/3 of root length. MX, Maxillary; M, Months; RR, Root Resorption; PA, Periapical.

##### Prevalence and severity

Prevalence of incisor root resorption ranged between 65.6% and 98.1%, depending on whether it was calculated per patient or per tooth. When calculating per patient, resorption ranged between 65.6% and 98.1% [32,35] whereas on a per tooth basis, resorption ranged between 72.9% and 94.2% [32,33,37,40].

In this review, mild to moderate root resorption is considered to be anything less than 1/3 of the original root length. Three studies reported little resorption (1.7% to 27.1%) following treatment [32,33,39]. One study [39] reported that 6.25% of the treated maxillary incisors resulted in severe root resorption where greater than 1/3 of the original root length was lost. Another study [34] reported 17.2% (5/29) of treated patients experience resorption of greater than 4 mm in at least one maxillary incisor. Each study classified root resorption differently; however, all reported that the majority of teeth experienced mild to moderate resorption following treatment.

##### Sex and age

The majority of studies had both male and female patients; however, only one [32] reported no difference in root resorption between the sexes. Five studies [32,33,35,36,39] examined patients in their teenage years (age ranged from 12.4 to 13.6 years), whereas there was one research [38] that examined an adult sample (age 25.4 years).

##### Treatment time

Six studies [32,34–36,38,39] reported treatment duration ranging from 22 to 38 months. One study [32] reported a weak to moderate positive correlation when duration of treatment in months was compared to apical root resorption (*r* = 0.434, **α =** 0.01).

##### Treatment mechanics

Different treatment mechanics were used to correct the class II division I malocclusion. DeShields [32] corrected the class II malocclusion without extractions and relied on the use of headgear and/or class II elastics. Studies by Hollender [33] and Liou [38] both relied on extraction of upper first premolars and applying en-masse retraction of the anterior segment using coils with or without miniscrews. In the study by Reukers [35], treatment ranged from non-extraction to extraction of two, three, or four premolars. Another study by Mavragani [40] compared straight wire to standard edgewise using extraction of premolars. Similarly, Taner [36] and Martins' [39] studies corrected the malocclusion with extraction of four first premolars and using headgear and/or class II elastics.

##### Risk of bias assessment

Of the eight selected reports, seven were retrospective studies [32–34,36–39] and one was a prospective study [35]. The methodological quality assessment tool showed low to moderate methodological quality with variance of 38.46% to 69.23% of the total scores (Table 8).

**Table 8:** **Methodological score of selected reports**

**Methodological quality criteria**	**DeShields** [32]	**Hollender et al.** [33]	**Eisel et al.** [34]	**Taner et al.** [36]	**Mavragani et al.** [40] and [37]	**Liou and Chang** [38]	**Martins et al.** [39]
Eligibility criteria - clearly described (✓), adequate (✓)	✓≠	≠≠	✘	✓≠	✓≠	✓≠	✓≠
Sample size - calculated (✓), adequate (✓)	✘✓	✘✘	✘✘	✘✓	✘✓	✘✓	✘✓
Randomization/consecutive selection - stated (✓)	✘	✘	✓	✘	✘	✘	✘
Blinding of assessor - stated (✓)	✘	✘	✘	✘	✘	✘	✘
Intervention details - clearly described (✓)	≠	≠	✘	✓	✓	✓	✓
Outcome measures - clearly described (✓)	✓	✓	✓	✓	✓	✓	✓
Selective reporting - avoided (✓)	✘	✓	✓	✓	✓	✓	✓
Adverse effects - described (✓)	✘	✘	✓	✘	≠	✘	✘
Data analysis - appropriate (✓)	≠	✓	✓	✓	✓	✓	✓
Point estimates and variability - exact *p* value (✓), variability measures, SD/CI (✓)	✘≠	✘✓	✘≠	✘✓	✓	✓	✓
Quality score (percentage of total)	38.46	42.30	42.30	57.69	69.23	65.38	65.38

Maximum number of ✓ = 13. ✓, fulfilled satisfactorily the methodological criteria (1 check point); ≠, fulfilled partially the methodological criteria (0.5 check point); ✘, did not fulfill the methodological criteria (0 check point).

##### Synthesis of results

Data pooling of the selected reports was not suitable because of methodological and clinical heterogeneity across studies [31].

### Discussion

This systematic review examined OIEARR of maxillary and mandibular incisors in non-surgical extraction/non-extraction class II division 1 treatments. Looking at the pyramid of evidence, no randomized controlled trials (RCT) and only limited prospective data concerning the impact of treatment on root resorption were available for this systematic review. Following an extensive search of the literature, eight studies were considered eligible. Given the lack of RCT in this systematic review, the studies included may have a higher risk of bias. Six of the eight studies treated patients with edgewise appliance only, whereas two of the eight studies compared edgewise to straight wire. The studies included in this systematic review made measurements of root resorption using radiographs (periapical or lateral cephalometric).

#### Prevalence and severity

The prevalence of root resorption in our review ranged between 65.6% and 98.1%. The maxillary incisors are often regarded as being most susceptible to root resorption [7,10,25] due to blunted or bottle-shaped root form [9,13]. Similarly, this review noted one study where resorption was reported in 72.9% of maxillary incisors compared to 34.7% of the remaining maxillary teeth [33]. When assessing if the central or lateral is more susceptible to resorption, studies by Mavragani [37] and DeShields [32] showed closer frequency of root resorption between the central and lateral incisors.

While root resorption of incisors from class II treatment appears to be quite prevalent, overall resorption appears to be mild to moderate. The studies included in this review reported mild to moderate resorption of root with one study [39] reporting severe root resorption in 6.25% (7/112) of the teeth. A literature review by Weltman [25] found that with panoramic or periapical radiographs, OIEARR is usually less than 2.5 mm, with severe resorption (>4 mm or >1/3 original root length) being seen in only 1% to 5% of the teeth. Another study [34] reported 17.2% (5/29) of treated patients experienced resorption of greater than 4 mm in at least one maxillary incisor. There is a possibility that the reported percentage of severe root resorption might be overestimated, and thus care should be taken when interpreting this value. Based on the information available in the current studies, severe root resorption in terms of percentage of teeth affected in treatment of class II malocclusions appears consistent with what is currently published in the literature for orthodontic treatment in general.

##### Sex and age

Sex has been reported to be a potential individual risk factor for root resorption. In our systematic review, only one study [32] reported no difference in root resorption between sexes. The other studies did not explicitly mention a difference in root resorption between sexes; this may suggest that no difference was found. This finding is in agreement with a number of other large-scale studies in the literature [9,10,28], suggesting that sex is an unlikely risk factor for root resorption.

It is often wondered if adults experience more root resorption than adolescents undergoing orthodontic treatment. From this review, it appears that treatment of younger patients produced a mild to moderate amount of root resorption. When looking at the adult population, similar mild to moderate resorption in terms of percentage of root resorption compared to original root and absolute amount of resorption was also reported. However, no study directly compared root changes between younger and older patient.

The study by Mavragani et al. [37] reported mild to moderate overall root resorption; however, the authors did report that roots that were incompletely developed before orthodontic treatment reached a greater length than those that were fully developed at the start of treatment. The authors hypothesized that there might be a mechanism whereby the immature teeth with open apexes protects the younger roots against resorption during orthodontic treatment and allows them to reach the normal root length when compared to untreated controls. One proposed concept suggested that teeth with open apex experience less severe pulp changes, thereby allowing for greater biological tolerance during treatment.

Collectively, the included studies suggest that chronological age at the start of treatment may not be a primary indicator of root resorption; however it is possible that these patients, although different in terms of chronological age, are identical in terms of degree of root formation. The study by Mavragani et al. [37] found that the age was significantly higher among patients showing root resorption (12.8 to 12.9 years) of the maxillary lateral incisors during treatment than among those showing root elongation (11.5 to 11.6 years).

##### Treatment time

Six studies [32,35,36,38,39] reported treatment duration ranging from 21.6 ± 4.8 months to 38 ± 20 months and all reported mild to moderate root resorption. A study by Segal et al. [26] concluded that one of the treatment-related causes of root resorption was treatment duration.

It is unclear in the literature whether treatment time is related to root resorption. Only one study [32] in this review compared root resorption to treatment time, which reported a weak to moderate positive correlation between duration of treatment and apical root resorption. This finding [32] support the notion that treatment time is related to root resorption as suggested by McFadden et al. [54]; however, it contradicts other studies which suggest that the two factors are unrelated [55,56].

It would have been interesting to determine how long patients were in active treatment as suggested by Segal et al. [26], since treatment duration could be inflated, despite limited times of activation if patients were missing appointments or if the clinicians preferred longer times between appointments. Unfortunately, this information was not available from the selected studies so such assessments were not done.

##### Root displacement

The study by DeShields et al. [32] reported weak to moderate correlation between anteroposterior apical displacement and root resorption. In addition, Liou and Chang [38] noted apical displacement (retraction 3.0 mm/intrusion 2.7 mm with miniscrews; retraction 1.3 mm/intrusion 2.5 mm without miniscrews) when using en-masse retraction of the anterior maxillary teeth using coils. In camouflage orthodontic treatment, it is conceivable that the incisors are subjected to large apical displacements that may lead to OIEARR. Clinicians should always be careful whenever displacing root apexes and should be aware that this type of movement might result in mild to moderate resorption.

##### Class II division 1 treatment mechanics

Treatment mechanics involved any of the following: extractions, non-extraction, straight wire, edgewise, class II elastics, headgear, functional appliances, and miniscrews to correct the malocclusion. Given the diverse range in techniques used to correct the malocclusion, what was interesting to note was that all these studies [32–39] consistently reported mild to moderate root resorption following treatment. It seems that treatment of class II malocclusions with any of the treatment strategies generally produces similar root resorption and the amount is similar to what is reported for orthodontic treatment of other types of malocclusions. The etiology of root resorption appears to be complex, so it is important for clinicians to recognize that there are many potential patient and treatment risk factors that may contribute to root resorption.

##### Measurement of root resorption

Assessment of root shape and length is an essential component of the initial diagnosis stage in orthodontics. Root resorption occurs tri-dimensionally (3D); however, most of the reported information in the literature relies on the use of a bi-dimensional radiographic image (2D). Ideally, a 3D image would provide the most accurate information. Collectively, the available studies have provided important insight into root resorption; however, the varying degree of magnification and the limitations of 2D imaging make the quantitative value of these radiographs questionable [25]. When attempting to evaluate apical root resorption using 2D imaging techniques (periapical, panoramic, ceph), the image shows superimposition of all the root structures, thus complicating the measure of root resorption [57]. In addition, the angulation between incisor and radiographic film as well as the amount of magnification can affect the images obtained, thus potentially impacting on the clinician’s ability to properly diagnose the case [57].

The studies included in this review relied on 2D imaging to determine the amount of root resorption. One study [36] measured root resorption using cephalometric radiographs. Incisor root lengths can be quite distorted and obscured with this imaging technique given the number of overlapping structures. Any root resorption information should be taken with caution. For completeness, this systematic review included a study [36] assessing root resorption using cephalometric radiographs, given its inherent limitations. Interestingly, root resorption reported using this technique reported similar severity as reported for periapical radiographs. Periapical images can be taken with either the parallel or bisecting technique. The parallel technique tends to be accurate and produces little magnification whereas the bisecting technique has more potential for image distortion. One study [35] reported using a non-standardized bisecting technique to obtain the image whereas the other studies did not specify which technique was used. Given the inherent potential for image distortion, it was surprising that only two studies [38,40] reported using a correction factor for distortion in their calculation of root resorption. While the data from this systematic review will provide some beneficial information, it is important to recognize that the available data has some inherent limitations.

Future studies would benefit from using CBCT to assess root resorption. This 3D imaging technique allows for slices of the root and eliminates superimposition of structures. Using this imaging technique, clinicians are better able to visualize and assess root resorption on any surface of the root. Future studies using this imaging technique may provide more accurate insight into the severity and prevalence of root resorption of maxillary incisors while also providing information for mandibular incisors since there is currently no data available. These findings highlight the importance for proper informed consent of the potential risk and impact of OIEARR.

## Conclusions

No randomized controlled trials and only limited prospective data concerning the impact of non-surgical class II treatment on root resorption were available. The current limited evidence suggests that comprehensive orthodontic treatment to correct class II division 1 malocclusions causes increased prevalence and severity of root resorption compared to pretreatment values the more the incisor roots are displaced and the longer this movement takes.

## Abbreviations

EARR: incisor external apical root resorption
OIEARR: orthodontically induced external apical root resorption

## References

[1] Owman-Moll P, Kurol J, Lundgren D (1996). The effects of a four-fold increased orthodontic force magnitude on tooth movement and root resorptions. An intra-individual study in adolescents. Eur J Orthod.

[2] Owman-Moll P, Kurol J, Lundgren D (1996). Effects of a doubled orthodontic force magnitude on tooth movement and root resorptions. An inter-individual study in adolescents. Eur J Orthod..

[3] Kurol J, Owman-Moll P (1998). Hyalinization and root resorption during early orthodontic tooth movement in adolescents. Angle Orthod.

[4] Harry MR, Sims MR (1982). Root resorption in bicuspid intrusion. A scanning electron microscope study. Angle Orthod.

[5] Lupi JE, Handelman CS, Sadowsky C (1996). Prevalence and severity of apical root resorption and alveolar bone loss in orthodontically treated adults. Am J Orthod Dentofacial Orthop.

[6] Levander E, Bajka R, Malmgren O (1998). Early radiographic diagnosis of apical root resorption during orthodontic treatment: a study of maxillary incisors. Eur J Orthod.

[7] Remington DN, Joondeph DR, Artun J, Riedel RA, Chapko MK (1989). Long-term evaluation of root resorption occurring during orthodontic treatment. Am J Orthod Dentofacial Orthop.

[8] Apajalahti S, Peltola JS (2007). Apical root resorption after orthodontic treatment – a retrospective study. Eur J Orthod.

[9] Mirabella AD, Artun J (1995). Risk factors for apical root resorption of maxillary anterior teeth in adult orthodontic patients. Am J Orthod Dentofacial Orthop.

[10] Sameshima GT, Sinclair PM (2001). Predicting and preventing root resorption: Part I. Diagnostic factors. Am J Orthod Dentofacial Orthop..

[11] Linge BO, Linge L (1983). Apical root resorption in upper anterior teeth. Eur J Orthod.

[12] Linge L, Linge BO (1991). Patient characteristics and treatment variables associated with apical root resorption during orthodontic treatment. Am J Orthod Dentofacial Orthop.

[13] Levander E, Malmgren O (1988). Evaluation of the risk of root resorption during orthodontic treatment: a study of upper incisors. Eur J Orthod.

[14] Janson GRDLCG, Martins DR, Henriques JF, De Freitas MR (1999). A radiographic comparison of apical root resorption after orthodontic treatment with 3 different fixed appliance techniques. Am J Orthod Dentofacial Orthop.

[15] Levander E, Malmgren O, Stenback K (1998). Apical root resorption during orthodontic treatment of patients with multiple aplasia: a study of maxillary incisors. Eur J Orthod.

[16] Consolaro A, Consolaro A (2005). Movimentação dentária induzida: biologia aplicada à prática clínica. Reabsorções dentárias nas especialidades clínicas.

[17] Harris EF, Hassankiadeh S, Harris JT (1993). Maxillary incisor crown-root relationships in different angle malocclusions. Am J Orthod Dentofacial Orthop.

[18] Al-Qawasmi RAHJJ, Everett ET, Flury L, Liu L, Foroud TM, Macri JV, Roberts WE (2003). Genetic predisposition to external apical root resorption. Am J Orthod Dentofacial Orthop.

[19] Brezniak NWA (1993). Root resorption after orthodontic treatment: part 2. Literature review. Am J Orthod Dentofacial Orthop.

[20] Fox N (2005). Longer Orthodontic treatment may result in greater external apical root resorption. Evid Based Dent.

[21] Killiany DM (1999). Root resorption caused by orthodontic treatment: an evidence-based review of literature. Seminars in Orthodontics.

[22] Brezniak NWA (2002). Orthodontically induced inflammatory root resorption: part II - the clinical aspects. Angle Orthod.

[23] Brezniak NWA (1993). Root resorption after orthodontic treatment: part I. Literature review. Am J Orthod Dentofacial Orthop..

[24] Pizzo G, Licata ME, Guiglia R, Giuliana G (2007). Root resorption and orthodontic treatment. Review of the literature. Minerva Stomatologica.

[25] Weltman B, Vig KW, Fields HW, Shanker S, Kaizar EE (2010). Root resorption associated with orthodontic tooth movement: a systematic review. Am J Orthod Dentofacial Orthop.

[26] Segal GR, Schiffman PH, Tuncay OC (2004). Meta analysis of the treatment-related factors of external apical root resorption. Orthod Craniofac Res.

[27] Franchi L, Baccetti T (2006). Prediction of individual mandibular changes induced by functional jaw orthopedics followed by fixed appliances in Class II patients. Angle Orthod.

[28] Kaley J, Phillips C (1991). Factors related to root resorption in edgewise practice. Angle Orthod.

[29] Liberati A, Altman DG, Tetzlaff J, Mulrow C, Gotzsche PC, Ioannidis JP, Clarke M, Devereaux PJ, Kleijnen J, Moher D (2009). The PRISMA statement for reporting systematic reviews and meta-analyses of studies that evaluate health care interventions: explanation and elaboration. J Clin Epidemiol.

[30] Moher D, Hopewell S, Schulz KF, Montori V, Gotzsche PC, Devereaux PJ, Elbourne D, Egger M, Altman DG, Consolidated Standards of Reporting Trials Group (2010). CONSORT 2010 explanation and elaboration: updated guidelines for reporting parallel group randomised trials. J Clin Epidemiol.

[31] Higgins JPT, Green S (2008). Cochrane handbook for systematic reviews of interventions Cochrane Collaboration.

[32] DeShields RW (1969). A study of root resorption in treated Class II. Division I malocclusions. Angle Orthod..

[33] Hollender L, Rönnerman A, Thilander B (1980). Root resorption, marginal bone support and clinical crown length in orthodontically treated patients. Eur J Orthod.

[34] Eisel A, Katsaros C, Berg R (1994). The course and results of the orthodontic treatment of 44 consecutively treated Class-II cases. Fortschritte der Kieferorthopadie.

[35] Reukers E, Sanderink G, Kuijpers-Jagtman A, van't Hof M (1998). Radiographic evaluation of apical root resorption with 2 different types of edgewise appliances. Results of a randomized clinical trial. J Orofac Orthop..

[36] Taner T, Ciger S, Sencift Y (1999). Evaluation of apical root resorption following extraction therapy in subjects with Class I and Class II malocclusions. Eur J Orthod.

[37] Mavragani M, Bøe OE, Wisth PJ, Selvig KA (2002). Changes in root length during orthodontic treatment: advantages for immature teeth. Eur J Orthod.

[38] Liou EJW, Chang PMH (2010). Apical root resorption in orthodontic patients with en-masse maxillary anterior retraction and intrusion with miniscrews. Am J Orthod Dentofacial Orthop.

[39] Martins DR, Tibola D, Janson G, Maria FRT (2012). Effects of intrusion combined with anterior retraction on apical root resorption. Eur J Orthod.

[40] Mavragani M, Vergari A, Selliseth NJ, Boe OE, Wisth PL (2000). A radiographic comparison of apical root resorption after orthodontic treatment with a standard edgewise and a straight-wire edgewise technique. Eur J Orthod.

[41] Goldson L, Henrikson CO (1975). Root resorption during Begg treatment; a longitudinal roentgenologic study. Am J Orthod.

[42] Lee RY, Artun J, Alonzo TA (1999). Are dental anomalies risk factors for apical root resorption in orthodontic patients?. Am J Orthod Dentofacial Orthop.

[43] McNab S, Battistutta D, Taverne A, Symons AL (2000). External apical root resorption following orthodontic treatment. Angle Orthod.

[44] Alwali SMM, Persson M (2000). Apical root resorption of upper first molars as related to anchorage system. Swed Dent J.

[45] Brin I, Tulloch JFC, Koroluk L, Philips C (2003). External apical root resorption in Class II malocclusion: a retrospective review of 1- versus 2-phase treatment. Am J Orthod Dentofacial Orthop.

[46] Segal GR, Schiffman PH, Tuncay OC (2004). Meta analysis of the treatment-related factors of external apical root resorption. Orthodontics & Craniofacial Research.

[47] Nasiopoulos AT, Athanasiou AE, Papadopoulos MA, Kolokithas G, Ioannidou I (2006). Premolar root changes following treatment with the banded Herbst appliance. J Orofac Orthop.

[48] Janson G, Nakamura A, de Freitas MR, Henriques JFC, Pinzan A (2007). Apical root resorption comparison between Frankel and eruption guidance appliances. Am J Orthod Dentofacial Orthop.

[49] Huang Y, Wang XX, Zhang J, Liu C (2010). Root shortening in patients treated with two-step and en masse space closure procedures with sliding mechanics. Angle Orthod.

[50] Weltman B, Vig KWL, Fields HW, Shanker S, Kaizar EE (2010). Root resorption associated with orthodontic tooth movement: a systematic review. Am J Orthod Dentofacial Orthop.

[51] Sunku R, Roopesh R, Kancherla P, Perumalla KK, Yudhistar PV, Reddy VS (2011). Quantitative digital subtraction radiography in the assessment of external apical root resorption induced by orthodontic therapy: a retrospective study. J Contemp Dent Pract.

[52] Kinzinger GSM, Savvaidis S, Gross U, Gulden N, Ludwig B, Lisson J (2011). Am J Orthod Dentofacial Orthop.

[53] Megat Abdul Wahab R, Md Dasor M, Senafi S, Abang Abdullah AA, Yamamoto Z, Jemain AA, Zainal Ariffin SH (2013). Crevicular alkaline phosphatase activity and rate of tooth movement of female orthodontic subjects under different continuous force applications. Int J Dent.

[54] McFadden WM, Engstrom C, Engstrom H, Anholm JM (1989). A study of the relationship between incisor intrusion and root shortening. Am J Orthod Dentofacial Orthop.

[55] VonderAhe G (1973). Postretention status of maxillary incisors with root-end resorption. Angle Orthod.

[56] Dermaut LR, De Munck A (1986). Apical root resorption of upper incisors caused by intrusive tooth movement: a radiographic study. Am J Orthod Dentofacial Orthop.

[57] Campos MJ, Silva KS, Gravina MA, Fraga MR, Vitral RW (2013). Apical root resorption: the dark side of the root. Am J Orthod Dentofacial Orthop.

